# Enhancing Methodological Rigor in Mobile Health Care Research

**DOI:** 10.2196/68243

**Published:** 2025-01-03

**Authors:** Shuhan Tang

**Affiliations:** 1 Duke Global Health Institute, Duke University, Durham, NC, United States; 2 Global Health Research Center (GHRC), Duke Kunshan University, No. 8 Duke Avenue, Kunshan, 215316, China, 86 051236657240

**Keywords:** letter to the editor, health care professionals, mobile health care, technical training, cross-sectional survey, mobile, China, web-based questionnaire, logistic regression, mhealth, mobile health

Tang et al’s recent study published in *JMIR Human Factors* [1], titled “The Use of Mobile Health Care Among Medical Professionals in the Sichuan-Chongqing Region: Cross-Sectional Survey Study,” captured my attention. Their analysis of mobile health (mHealth) device use and influencing factors, using chi-square and multivariable logistic regression analyses, revealed a significant association between age and mHealth use. This study provides valuable insights from China’s western region.

However, I would like to offer a few comments and suggestions that I believe would further enhance the study’s methodology and findings.

First, the study’s reliance on a web-based questionnaire may introduce potential selection bias, as respondents, by nature of accessing web-based surveys, may be more likely to use digital devices and have a stronger interest in digital health in general. A more diverse, mixed-methods approach to questionnaire distribution could help mitigate this potential self-selection toward mHealth use and prevent the exclusion of individuals with limited experience or interest in mHealth.

Second, the study could benefit from a standardized and rigorous methodological framework for survey design and reporting. For instance, categorizing age into three broad groups may overlook essential trends. A finer measurement scale for satisfaction and usage, such as a Likert scale, could provide deeper insights into health care professionals’ attitudes. Furthermore, for questions where “uncertain” responses outweigh “yes” or “no” responses, a qualitative or mixed-methods research approach could yield a more nuanced understanding of the underlying reasons. Finally, incorporating years of work experience as a variable could add valuable insights, given its potential correlation with age. Unlike professional titles, years of work experience could provide a more direct measure of professional tenure, potentially enriching the data analysis.

Third, ethical considerations of this study merit further attention. Identifying specific hospitals in the report may compromise confidentiality. Additionally, the exemption of review by an institutional review board (IRB) raises concerns, as the study involves gathering potentially sensitive information from human participants (ie, health care professionals) regarding personal perspectives, workflows, and technology use. Such studies often warrant an ethics board review to protect participant privacy, minimize psychological or social risks, and ensure adherence to ethical standards [2].

Fourth, before conducting logistic regression analysis, normality tests on both independent and dependent variables are recommended to confirm the validity of the chosen statistical methods [3]. If data are not normally distributed, adjustments should be considered.

Lastly, as noted in the “Limitations” section [1], the reliance on convenience sampling may affect the study’s generalizability. Only public, district-level hospitals were included, excluding primary and tertiary institutions—this limits the representation of China’s complete health care hierarchy in the analysis. Furthermore, only urban areas were sampled, overlooking rural populations. Women comprised 77.1% of the study sample, raising questions about gender representation. A multi-level sampling approach would likely yield a more comprehensive and representative dataset.

To conclude, I would like to highlight that these feedback points are not to challenge the integrity of the authors’ work. Instead, I hope they can contribute to ongoing discussions on mHealth research and the development of robust methodologies in this field.

## Abbreviations

IRB: institutional review board
mHealth: mobile health
